# Supplementary material for: Integrating Association Mapping, Linkage Mapping, Fine Mapping with RNA Seq Conferring Seedling Vigor Improvement for Successful Crop Establishment in Deep Sown Direct-Seeded Rice
Source: Rice (N Y). 2023 Oct 17;16:46. doi: 10.1186/s12284-023-00665-w (PMC10581981; doi:10.1186/s12284-023-00665-w)
Supplement: Supplementary file 1 — Additional file 1. Fig. S1. The QQ plots for different seedling vigor, grain yield and yield associated traits across years. Fig. S2. The frequency distribution curve for different seedling vigor, grain yield and yield associated traits across years. Fig. S3. QTL likelihood curves of LOD scores for (A) root length (B) shoot length at 6 cm sowing depth considering pooled mean analysis across years on chr 7 in PR126 x IRGC 128442 mapping population. Fig. S4. QTL likelihood curves of LOD scores for (A) days to 50% flowering (DTF) (B) Plant height (PHT) and (C) grain yield (GY) under direct-seeded rice field conditions considering pooled mean analysis across years on chr 7 in PR126 x IRGC 128442 mapping population. Fig. S5. The role of validated candidate genes associated with mesocotyl elongation in different (A) biological processes, (B) molecular functions and (C) cellular components at three different time points (5, 10 and 15 days after sowing) and two different sowing depths (4 cm as control and 10 cm as treatment). Table S1. Details on the chromosome wise number of SNPs, map distance and average marker distance. Table S2. Summary of the alignment of 106 samples aligned to the Oryza sativa reference genome. [file 12284_2023_665_MOESM1_ESM.docx]

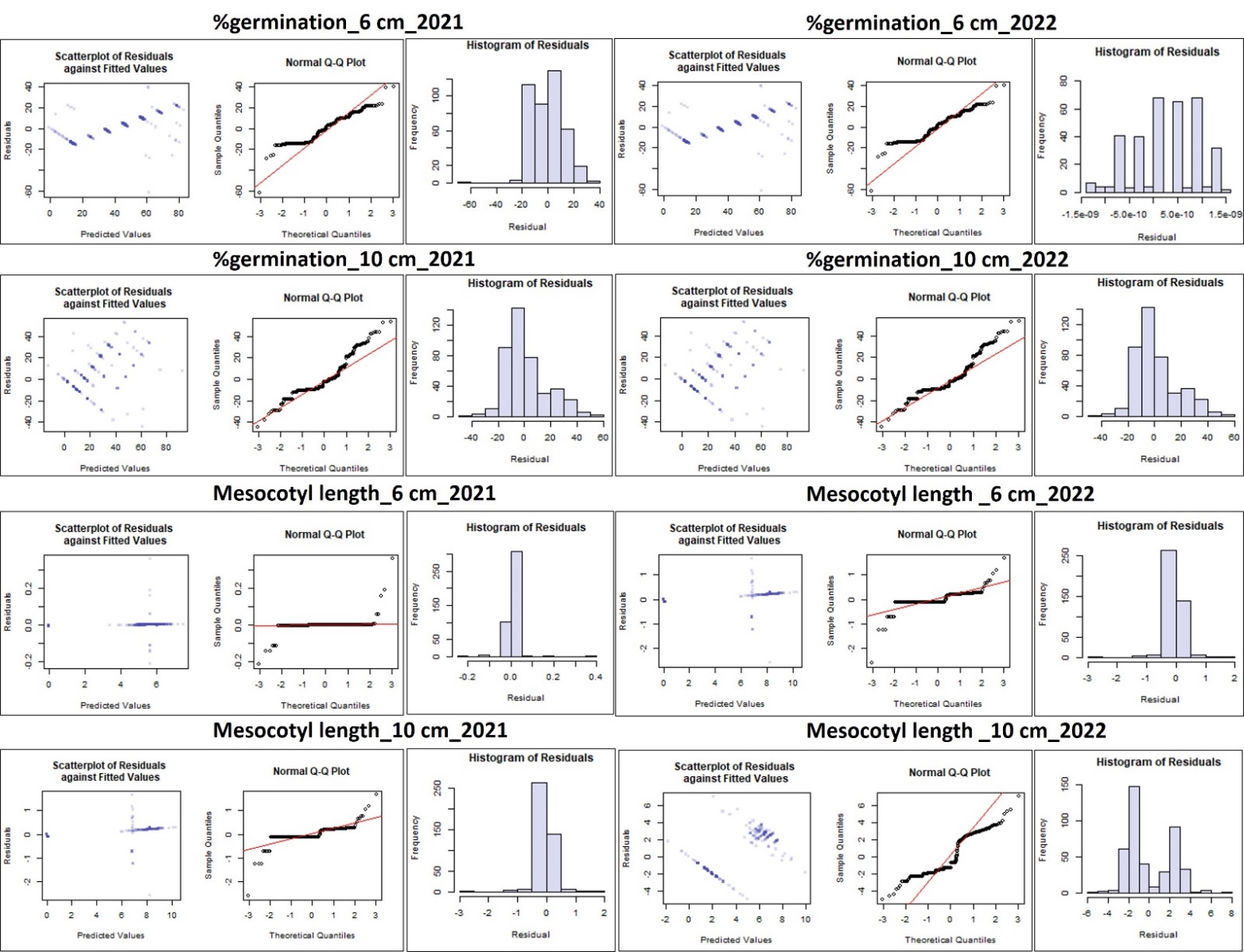

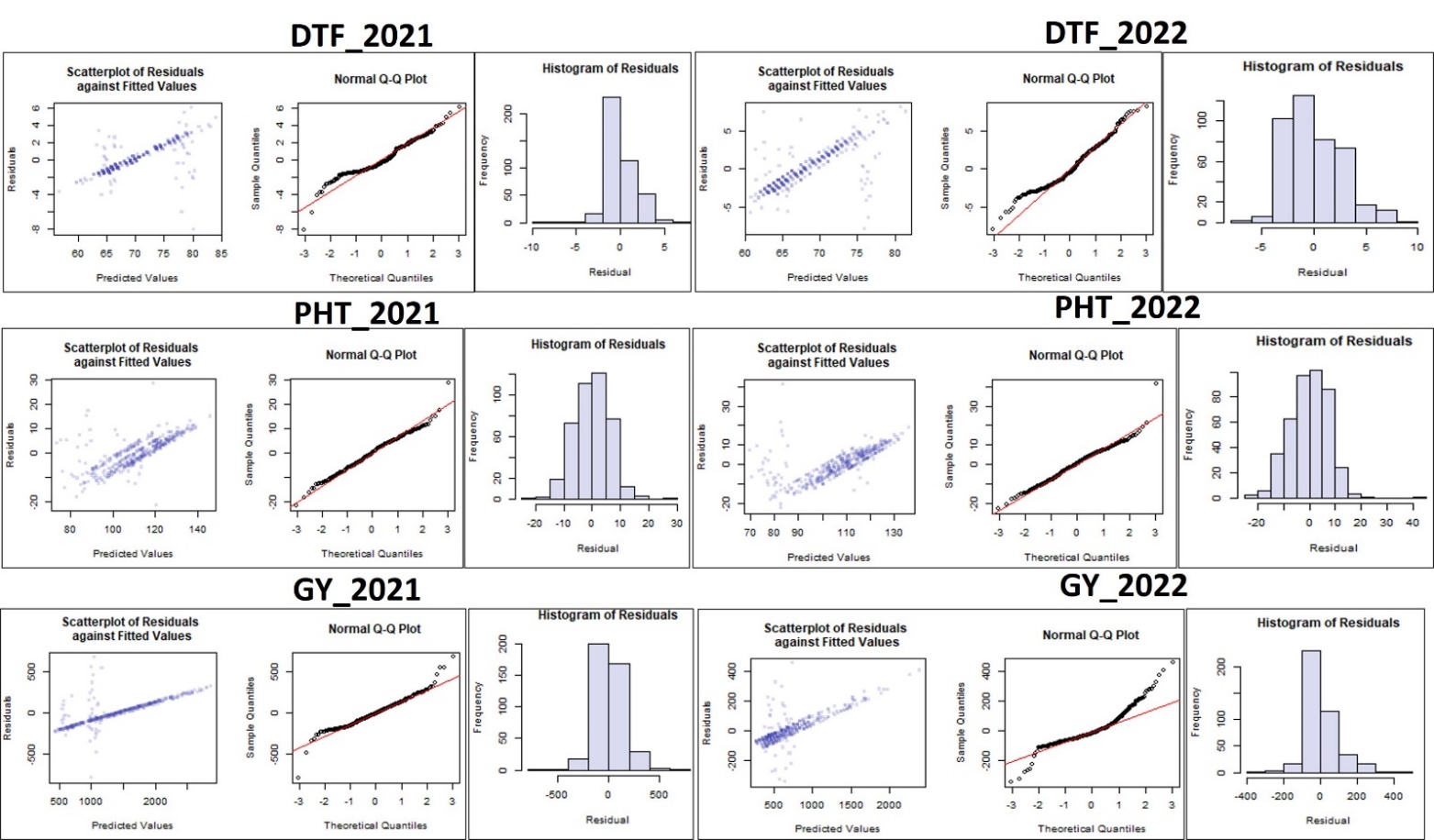


**Fig. S1.** The QQ plots for different seedling vigor, grain yield and yield associated traits across years


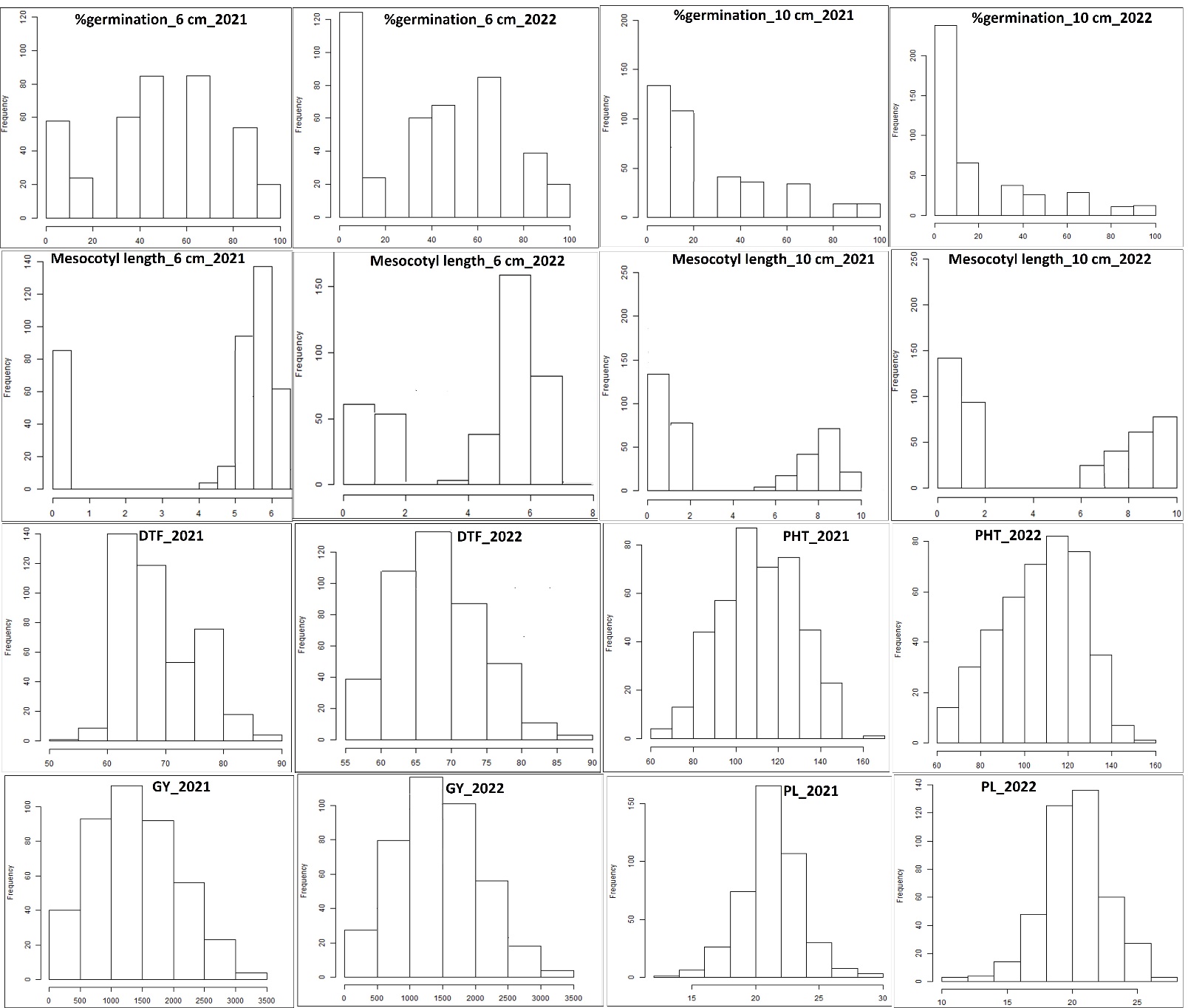


**Fig. S2.** The frequency distribution curve for different seedling vigor, grain yield and yield associated traits across years


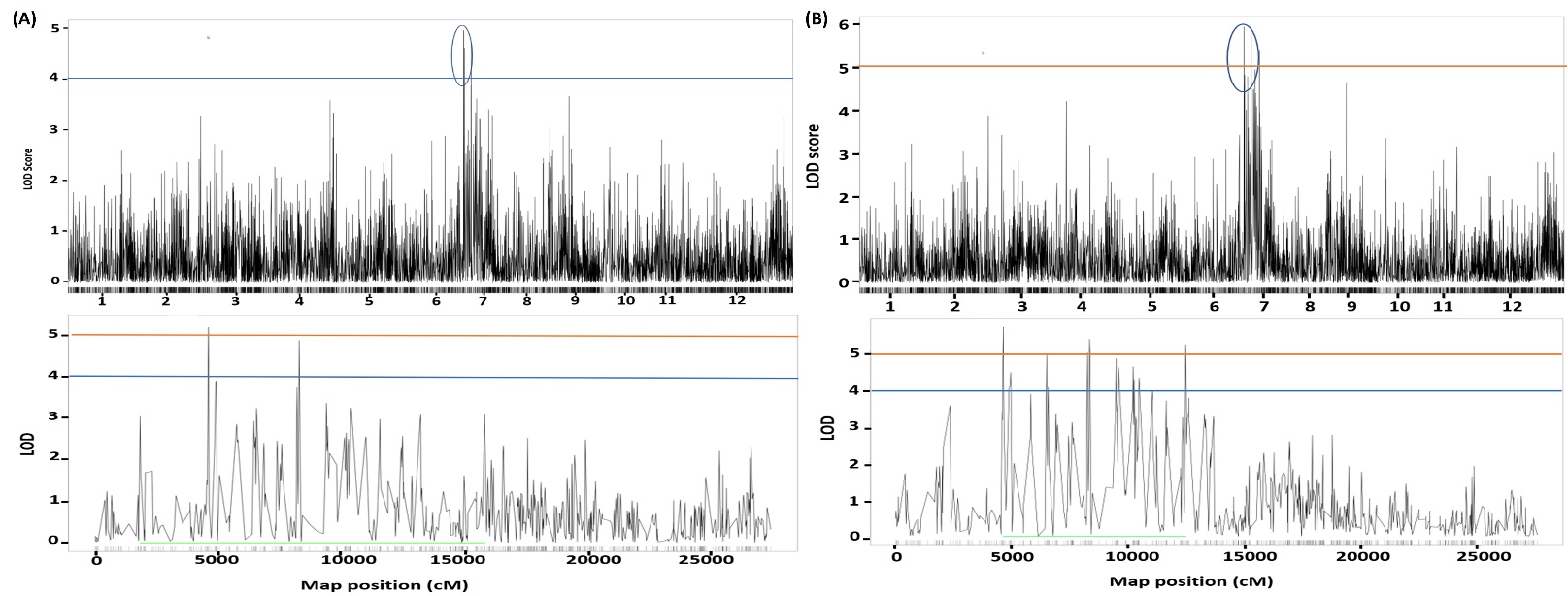


**Fig. S3.** QTL likelihood curves of LOD scores for (A) root length (B) shoot length at 6 cm sowing depth considering pooled mean analysis across years on chr 7 in PR126 x IRGC 128442 mapping population


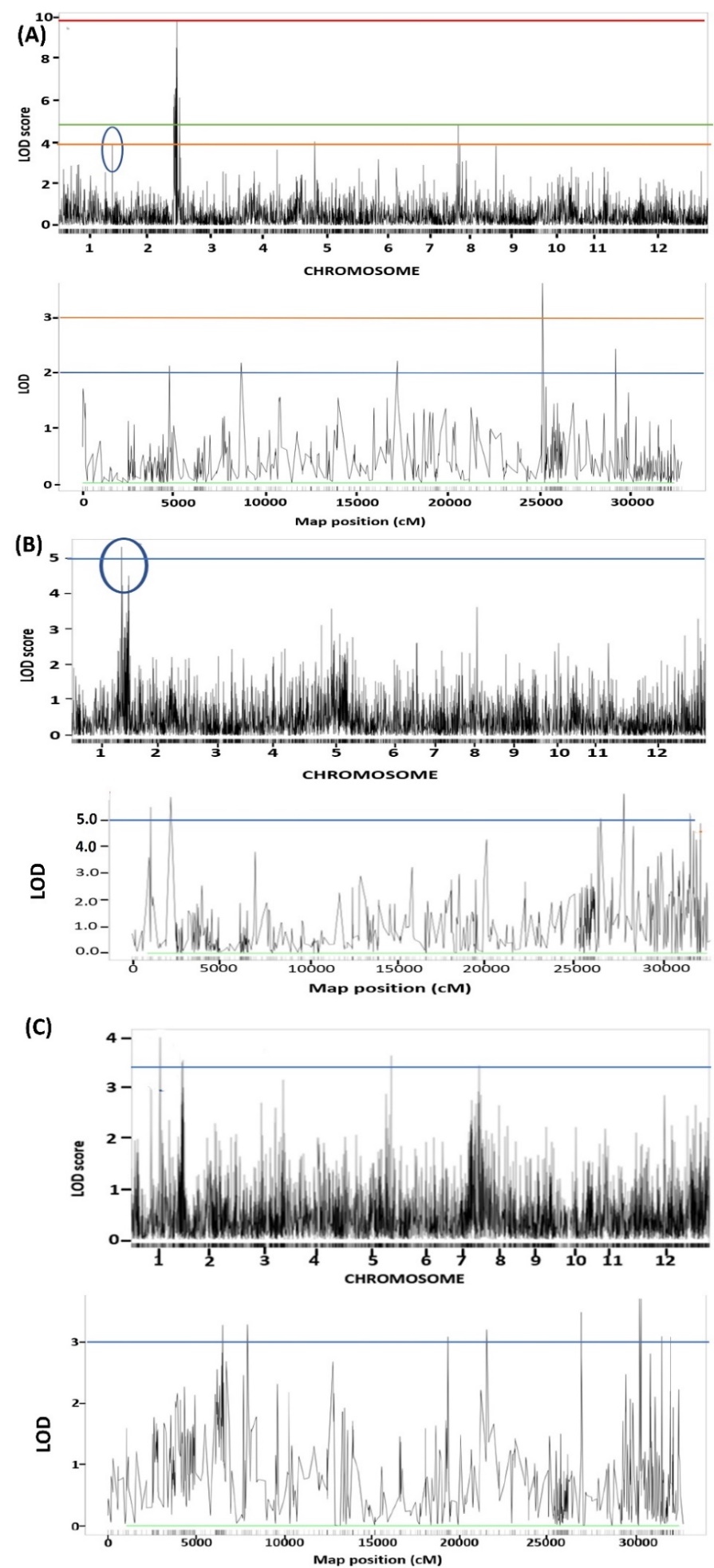


**Fig. S4.** QTL likelihood curves of LOD scores for (A) days to 50% flowering (DTF) (B) Plant height (PHT) and (C) grain yield (GY) under direct-seeded rice field conditions considering pooled mean analysis across years on chr 7 in PR126 x IRGC 128442 mapping population


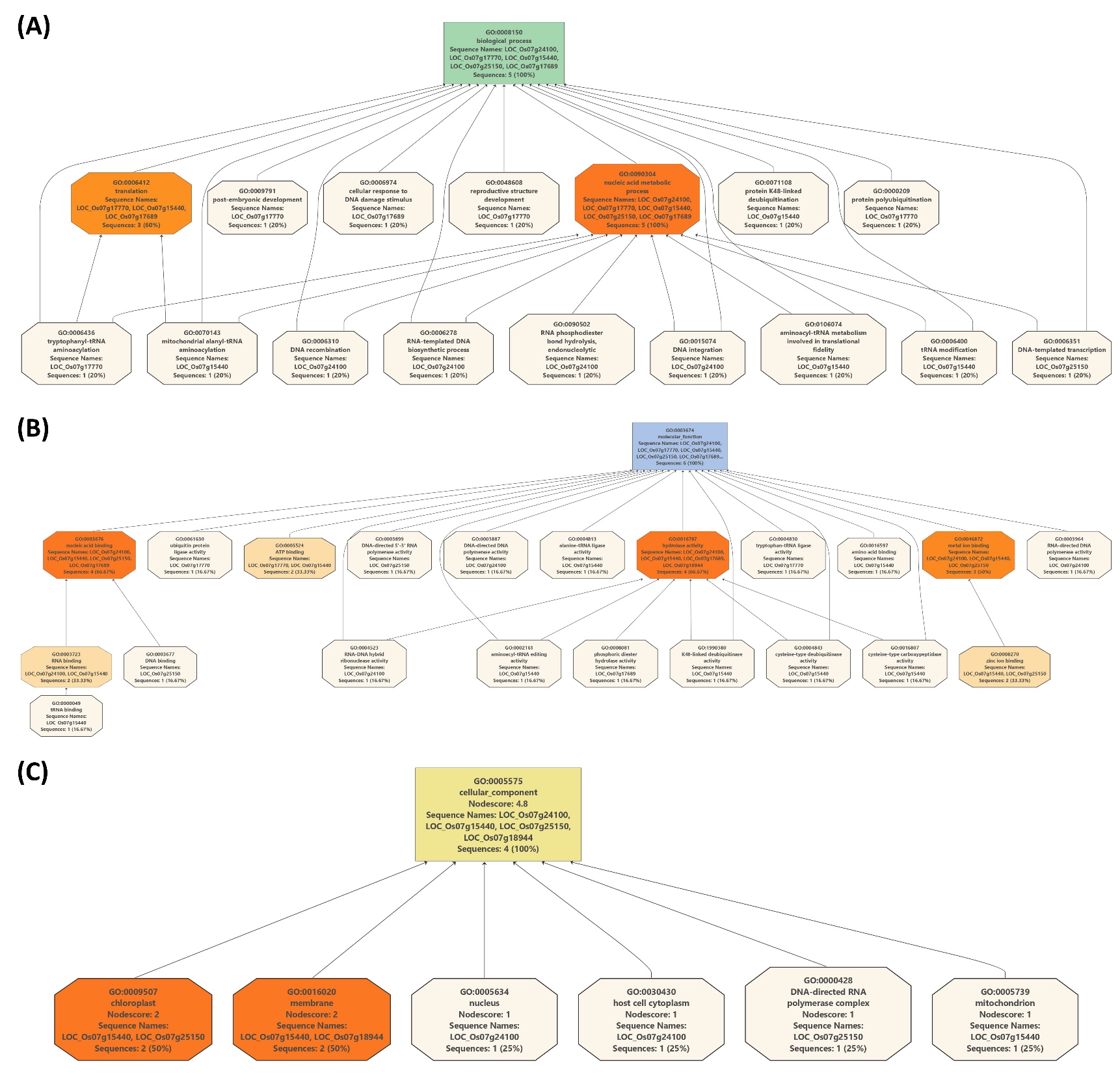


**Fig. S5.** The role of validated candidate genes associated with mesocotyl elongation in different (A) biological processes, (B) molecular functions and (C) cellular components at three different time points (5, 10 and 15 days after sowing) and two different sowing depths (4 cm as control and 10 cm as treatment).

**Table S1.** Details on the chromosome wise number of SNPs, map distance and average marker distance

| **LG_name** | **Number of**  **markers** | **Map distance (cM)** | **Average marker distance (cM)** |
| --- | --- | --- | --- |
| Chr1 | 885 | 172.2 | 0.19 |
| Chr2 | 908 | 114.965 | 0.13 |
| Chr3 | 1127 | 104.986 | 0.09 |
| Chr4 | 486 | 119.242 | 0.25 |
| Chr5 | 1454 | 101.972 | 0.07 |
| Chr6 | 351 | 166.881 | 0.48 |
| Chr7 | 807 | 107.763 | 0.13 |
| Chr8 | 395 | 167.455 | 0.42 |
| Chr9 | 953 | 135.299 | 0.14 |
| Chr10 | 440 | 146.972 | 0.33 |
| Chr11 | 499 | 97.908 | 0.21 |
| Chr12 | 1993 | 125.966 | 0.06 |
| Total number of Markers | 10298 |  |  |
| Average Intermarker Distance (cM) | 0.2075 |  |  |

**Table S2.** Summary of the alignment of 106 samples aligned to the *Oryza sativa* reference genome

| **Sample** | | **Total features** | | **Aligned** | **Aligned_**  **Percentage** | **Not aligned** | **Not_aligned_**  **Percentage** |
| --- | --- | --- | --- | --- | --- | --- | --- |
| PR126, Stage1, 4cm, BR1, TR1 | | 15755390 | | 13669777 | 86.76 | 2085613 | 13.24 |
| PR126, Stage1, 4cm, BR1, TR2 | | 15764921 | | 13678812 | 86.77 | 2086109 | 13.23 |
| PR126, Stage1, 4cm, BR1, TR3 | | 15741340 | | 13656715 | 86.76 | 2084625 | 13.24 |
| IRGC 128442, Stage1, 4cm, BR3, TR1 | | 16482986 | | 15849684 | 96.16 | 633302 | 3.84 |
| IRGC 128442, Stage1, 4cm, BR3, TR2 | | 16383201 | | 15757192 | 96.18 | 626009 | 3.82 |
| IRGC 128442, Stage1, 4cm, BR3, TR3 | | 16476788 | | 15849883 | 96.2 | 626905 | 3.8 |
| IRGC 128442, Stage2, 10cm, BR1, TR1 | | 17137195 | | 16223440 | 94.67 | 913755 | 5.33 |
| IRGC 128442, Stage2, 10cm, BR1, TR2 | | 16989377 | | 16095342 | 94.74 | 894035 | 5.26 |
| IRGC 128442, Stage2, 10cm, BR1, TR3 | | 17276105 | | 16377363 | 94.8 | 898742 | 5.2 |
| IRGC 128442, Stage2, 4cm, BR1, TR1 | | 16134902 | | 14385860 | 89.16 | 1749042 | 10.84 |
| IRGC 128442, Stage2, 4cm, BR1, TR2 | | 16046850 | | 14320583 | 89.24 | 1726267 | 10.76 |
| IRGC 128442, Stage2, 4cm, BR1, TR3 | | 16187449 | | 14463275 | 89.35 | 1724174 | 10.65 |
| IRGC 128442, Stage2, 10cm, BR2, TR1 | | 15633750 | | 15239636 | 97.48 | 394114 | 2.52 |
| IRGC 128442, Stage2, 10cm, BR2, TR2 | | 17434892 | | 16989331 | 97.44 | 445561 | 2.56 |
| IRGC 128442, Stage2, 10cm, BR2, TR3 | | 15750356 | | 15351536 | 97.47 | 398820 | 2.53 |
| IRGC 128442, Stage2, 4cm, BR2, TR1 | | 19591253 | | 19451748 | 99.29 | 139505 | 0.71 |
| IRGC 128442, Stage2, 4cm, BR2, TR2 | | 19557991 | | 19419221 | 99.29 | 138770 | 0.71 |
| IRGC 128442, Stage2, 4cm, BR2, TR3 | | 19546725 | | 19408545 | 99.29 | 138180 | 0.71 |
| PR126, Stage2, 4cm, BR1, TR1 | | 18193697 | | 16754322 | 92.09 | 1439375 | 7.91 |
| PR126, Stage2, 4cm, BR1, TR2 | | 18054242 | | 16633974 | 92.13 | 1420268 | 7.87 |
| PR126, Stage2, 4cm, BR1, TR3 | | 17934809 | | 16540872 | 92.23 | 1393937 | 7.77 |
| PR126, Stage2, 10cm, BR1, TR1 | | 20302051 | | 19550850 | 96.3 | 751201 | 3.7 |
| PR126, Stage2, 10cm, BR1, TR2 | | 20125781 | | 19380048 | 96.29 | 745733 | 3.71 |
| PR126, Stage2, 10cm, BR1, TR3 | | 20364477 | | 19604632 | 96.27 | 759845 | 3.73 |
| IRGC 128442, Stage2, 10cm, BR3, TR1 | | 21200417 | | 21081276 | 99.44 | 119141 | 0.56 |
| IRGC 128442, Stage2, 10cm, BR3, TR2 | | 20942622 | | 20823720 | 99.43 | 118902 | 0.57 |
| IRGC 128442, Stage2, 10cm, BR3, TR3 | | 21386107 | | 21261847 | 99.42 | 124260 | 0.58 |
| PR126, Stage2, 4cm, BR2, TR1 | | 18318820 | | 18113546 | 98.88 | 205274 | 1.12 |
| PR126, Stage2, 4cm, BR2, TR2 | | 18326515 | | 18120494 | 98.88 | 206021 | 1.12 |
| PR126, Stage2, 4cm, BR2, TR3 | | 18340838 | | 18132492 | 98.86 | 208346 | 1.14 |
| PR126, Stage1, 10cm, BR1, TR1 | | 15994439 | | 15726172 | 98.32 | 268267 | 1.68 |
| PR126, Stage1, 10cm, BR1, TR2 | | 15848333 | | 15581210 | 98.31 | 267123 | 1.69 |
| PR126, Stage1, 10cm, BR1, TR3 | | 15714713 | | 15450969 | 98.32 | 263744 | 1.68 |
| IRGC 128442, Stage2, 4cm, BR3, TR1 | | 14868491 | | 14039745 | 94.43 | 828746 | 5.57 |
| IRGC 128442, Stage2, 4cm, BR3, TR2 | | 14974888 | | 14139563 | 94.42 | 835325 | 5.58 |
| IRGC 128442, Stage2, 4cm, BR3, TR3 | | 15064797 | | 14221980 | 94.41 | 842817 | 5.59 |
| PR126, Stage2, 4cm, BR3, TR1 | | 18948512 | | 16544890 | 87.31 | 2403622 | 12.69 |
| PR126, Stage2, 4cm, BR3, TR2 | | 18839208 | | 16452811 | 87.33 | 2386397 | 12.67 |
| PR126, Stage2, 4cm, BR3, TR3 | | 18750611 | | 16391190 | 87.42 | 2359421 | 12.58 |
| PR126, Stage2, 4cm, BR4, TR1 | | 17979455 | | 17392691 | 96.74 | 586764 | 3.26 |
| PR126, Stage2, 4cm, BR4, TR2 | | 17954896 | | 17362321 | 96.7 | 592575 | 3.3 |
| PR126, Stage2, 4cm, BR4, TR3 | | 17960892 | | 17369052 | 96.7 | 591840 | 3.3 |
| PR126, Stage2, 10cm, BR2, TR1 | | 15739469 | | 14792167 | 93.98 | 947302 | 6.02 |
| PR126, Stage2, 10cm, BR2, TR2 | | 17931850 | | 16854191 | 93.99 | 1077659 | 6.01 |
| PR126, Stage2, 10cm, BR2, TR3 | | 15742186 | | 14795369 | 93.99 | 946817 | 6.01 |
| IRGC 128442, Stage3, 10cm, BR1, TR1 | | 20495117 | | 19553175 | 95.4 | 941942 | 4.6 |
| IRGC 128442, Stage3, 10cm, BR1, TR2 | | 20686590 | | 19729549 | 95.37 | 957041 | 4.63 |
| IRGC 128442, Stage3, 10cm, BR1, TR3 | | 20887619 | | 19917888 | 95.36 | 969731 | 4.64 |
| IRGC 128442, Stage3, 10cm, BR2, TR1 | | 18246370 | | 16544860 | 90.67 | 1701510 | 9.33 |
| IRGC 128442, Stage3, 10cm, BR2, TR2 | | 18137111 | | 16450017 | 90.7 | 1687094 | 9.3 |
| IRGC 128442, Stage3, 10cm, BR2, TR3 | | 18404946 | | 16655499 | 90.49 | 1749447 | 9.51 |
| PR126, Stage3, 4cm, BR1, TR1 | | 19787502 | | 18763575 | 94.83 | 1023927 | 5.17 |
| PR126, Stage3, 4cm, BR1, TR2 | | 19395089 | | 18389836 | 94.82 | 1005253 | 5.18 |
| PR126, Stage3, 4cm, BR1, TR3 | | 20051636 | | 19012146 | 94.82 | 1039490 | 5.18 |
| PR126, Stage3, 4cm, BR2, TR1 | | 17849260 | | 16157836 | 90.52 | 1691424 | 9.48 |
| PR126, Stage3, 4cm, BR2, TR2 | | 17709735 | | 16036434 | 90.55 | 1673301 | 9.45 |
| PR126, Stage3, 4cm, BR2, TR3 | | 17975828 | | 16289702 | 90.62 | 1686126 | 9.38 |
| IRGC 128442, Stage3, 10cm, BR3, TR1 | | 18547589 | | 16340447 | 88.1 | 2207142 | 11.9 |
| IRGC 128442, Stage3, 10cm, BR3, TR2 | | 18688014 | | 16460220 | 88.08 | 2227794 | 11.92 |
| IRGC 128442, Stage3, 10cm, BR3, TR3 | | 18336110 | | 16144635 | 88.05 | 2191475 | 11.95 |
| IRGC 128442, Stage3, 4cm, BR1, TR1 | | 19989324 | | 17725188 | 88.67 | 2264136 | 11.33 |
| IRGC 128442, Stage3, 4cm, BR1, TR2 | | 19539844 | | 17316978 | 88.62 | 2222866 | 11.38 |
| IRGC 128442, Stage3, 4cm, BR1, TR3 | | 20388994 | | 18070706 | 88.63 | 2318288 | 11.37 |
| PR126, Stage1, 4cm, BR2, TR1 | | 18664275 | | 16729149 | 89.63 | 1935126 | 10.37 |
| PR126, Stage1, 4cm, BR2, TR2 | | 18557288 | | 16622961 | 89.58 | 1934327 | 10.42 |
| PR126, Stage1, 4cm, BR2, TR3 | | 18766377 | | 16805484 | 89.55 | 1960893 | 10.45 |
| PR126, Stage3, 4cm, BR3, TR1 | | 19149492 | | 17289306 | 90.29 | 1860186 | 9.71 |
| PR126, Stage3, 4cm, BR3, TR2 | | 19147208 | | 17267233 | 90.18 | 1879975 | 9.82 |
| PR126, Stage3, 4cm, BR3, TR3 | | 19087819 | | 17215390 | 90.19 | 1872429 | 9.81 |
| IRGC 128442, Stage3, 4cm, BR2, TR1 | | 18553232 | | 16118208 | 86.88 | 2435024 | 13.12 |
| IRGC 128442, Stage3, 4cm, BR2, TR2 | | 18434701 | | 16009116 | 86.84 | 2425585 | 13.16 |
| IRGC 128442, Stage3, 4cm, BR2, TR3 | | 18303213 | | 15889797 | 86.81 | 2413416 | 13.19 |
| PR126, Stage3, 10cm, BR1, TR1 | | 16726805 | | 16171012 | 96.68 | 555793 | 3.32 |
| PR126, Stage3, 10cm, BR1, TR2 | | 16618310 | | 16056379 | 96.62 | 561931 | 3.38 |
| PR126, Stage3, 10cm, BR1, TR3 | | 16805208 | | 16236872 | 96.62 | 568336 | 3.38 |
| PR126, Stage3, 10cm, BR2, TR1 | | 17685850 | | 17123881 | 96.82 | 561969 | 3.18 |
| PR126, Stage3, 10cm, BR2, TR2 | | 17495427 | | 16933633 | 96.79 | 561794 | 3.21 |
| PR126, Stage3, 10cm, BR2, TR3 | | 17763821 | | 17193118 | 96.79 | 570703 | 3.21 |
| IRGC 128442, Stage3, 4cm, BR3, TR1 | | 19947202 | | 18673563 | 93.61 | 1273639 | 6.39 |
| IRGC 128442, Stage3, 4cm, BR3, TR2 | | 19794069 | | 18526543 | 93.6 | 1267526 | 6.4 |
| IRGC 128442, Stage3, 4cm, BR3, TR3 | | 20015620 | | 18727700 | 93.57 | 1287920 | 6.43 |
| IR5796, Stage1, 10cm, BR3, TR1 | | 14345978 | | 13612082 | 94.88 | 733896 | 5.12 |
| IR5796, Stage1, 10cm, BR3, TR2 | | 14367056 | | 13628901 | 94.86 | 738155 | 5.14 |
| IR5796, Stage1, 10cm, BR3, TR3 | | 14916942 | | 14458536 | 96.93 | 458406 | 3.07 |
| IR5796, Stage1, 10cm, BR1, TR1 | | 15708871 | | 15106280 | 96.16 | 602591 | 3.84 |
| IR5796, Stage1, 10cm, BR1, TR2 | | 15664971 | | 15069951 | 96.2 | 595020 | 3.8 |
| PR126, Stage1, 10cm, BR3, TR1 | | 14026479 | | 11604049 | 82.73 | 2422430 | 17.27 |
| PR126, Stage1, 10cm, BR3, TR2 | | 14085027 | | 11659369 | 82.78 | 2425658 | 17.22 |
| PR126, Stage1, 4cm, BR3, TR1 | | 13592994 | | 12698977 | 93.42 | 894017 | 6.58 |
| PR126, Stage1, 4cm, BR3, TR2 | | 13520409 | | 12631307 | 93.42 | 889102 | 6.58 |
| PR126, Stage1, 4cm, BR3, TR3 | | 13580656 | | 12689988 | 93.44 | 890668 | 6.56 |
| PR126, Stage1, 10cm, BR2, TR1 | | 15209644 | | 14222058 | 93.51 | 987586 | 6.49 |
| PR126, Stage1, 10cm, BR2, TR2 | | 15061362 | | 14083660 | 93.51 | 977702 | 6.49 |
| PR126, Stage1, 10cm, BR2, TR3 | | 15284257 | | 14284359 | 93.46 | 999898 | 6.54 |
| IRGC 128442, Stage1, 4cm, BR1, TR1 | | 16618173 | | 16033419 | 96.48 | 584754 | 3.52 |
| IRGC 128442, Stage1, 4cm, BR1, TR2 | | 16475351 | | 15893374 | 96.47 | 581977 | 3.53 |
| IRGC 128442, Stage1, 4cm, BR1, TR3 | | 16581305 | | 15993338 | 96.45 | 587967 | 3.55 |
| IRGC 128442, Stage1, 10cm, BR2, TR1 | | 17503416 | | 16754504 | 95.72 | 748912 | 4.28 |
| IRGC 128442, Stage1, 10cm, BR2, TR2 | | 17250081 | | 16516863 | 95.75 | 733218 | 4.25 |
| IRGC 128442, Stage1, 10cm, BR2, TR3 | | 17686986 | | 16937894 | 95.76 | 749092 | 4.24 |
| IRGC 128442, Stage1, 4cm, BR2, TR1 | | 15993684 | | 14686092 | 91.82 | 1307592 | 8.18 |
| IRGC 128442, Stage1, 4cm, BR2, TR2 | | 16030556 | | 14712477 | 91.78 | 1318079 | 8.22 |
| IRGC 128442, Stage1, 4cm, BR2, TR3 | | 15915800 | | 14605881 | 91.77 | 1309919 | 8.23 |
| Average | | 17531526.02 | |  | 93.459 |  | 6.541 |
| Stage1= 5 days after sowing | |  |  |  |  |  |  |
| Stage2= 10 days after sowing | |  |  |  |  |  |  |
| Stage3= 15 days after sowing | |  |  |  |  |  |  |
| BR= Biological Replicate | |  |  |  |  |  |  |
| TR= Technical Replicate | |  |  |  |  |  |  |
